# National Trends for Temporary Mechanical Circulatory Support Utilization in Patients With Cardiogenic Shock From Decompensated Chronic Heart Failure: Incidence, Predictors, Outcomes, and Cost

**DOI:** 10.1016/j.jscai.2023.101177

**Published:** 2023-12-04

**Authors:** Aroosa Malik, Tanima Basu, Gabriella VanAken, Vikas Aggarwal, Ran Lee, Ahmad Abdul-Aziz, Edo Y. Birati, Mir Babar Basir, Brahmajee K. Nallamothu, Supriya Shore

**Affiliations:** a Division of Cardiology, Department of Internal Medicine, University of Michigan, Ann Arbor, Michigan; b University of Michigan Medical School, Ann Arbor, Michigan; c Division of Cardiology, Department of Internal Medicine, Henry Ford Hospital, Detroit, Michigan; d Division of Cardiology, Department of Internal Medicine, Cleveland Clinic, Cleveland, Ohio; e Division of Cardiology, Department of Internal Medicine, Inova Heart and Vascular Institute, Falls Church, Virginia; f Division of Cardiology, Department of Internal Medicine, Poriya Medical Center, Bar-Ilan University, Israel; g Institute of Healthcare Policy and Innovation, University of Michigan, Ann Arbor, Michigan

**Keywords:** cardiogenic shock, extracorporeal membrane oxygenation, heart failure, intra-aortic balloon pump, Impella, temporary mechanical circulatory support

## Abstract

**Background:**

Trends in temporary mechanical circulatory support (tMCS) use with associated outcomes and cost in cardiogenic shock secondary to decompensated chronic heart failure (HF-CS) remains poorly understood. We describe trends in tMCS use, associated outcomes, and cost in HF-CS.

**Methods:**

We included adults enrolled in a national insurance claims dataset with HF-CS who received intra-aortic balloon pump (IABP), Impella, or extracorporeal membrane oxygenation (ECMO) without acute coronary syndrome, or postcardiotomy shock. We identified predictors of device use, associated outcomes, and inflation-adjusted costs.

**Results:**

We studied 2722 HF-CS patients receiving tMCS: 1799 (66%) male, 1771 (65%) White, and 1836 (67%) with ischemic cardiomyopathy. Rate of tMCS use increased from 2010-2019. Impella use showed the largest increase (Δ+344%), followed by ECMO (Δ+112%). Patients receiving ECMO had a higher comorbidity burden, and patients receiving IABP were more likely to have valvular heart disease. Compared with IABP, 30-day mortality rate was no different for Impella (adjusted odds ratio, 1.24; 95% CI, 0.93-1.66) but was higher with ECMO (adjusted odds ratio, 3.08; 95% CI, 2.22-4.27). Adjusted hospitalization cost was highest for ECMO (median, $191,079 [IQR, $165,760-$239,373]), followed by Impella (median, $142,518 [IQR, $126,845-$179,938]), and IABP (median, $132,060 [IQR, $113,794-$160,244]). We observed a linear association between price standardized cost-quartile and complications, but not for 30-day mortality.

**Conclusions:**

The use of Impella and ECMO is increasing with an associated cost increase. The use of ECMO coincided with higher 30-day mortality compared with IABP in HF-CS. These findings likely reflect increasing disease severity and evolving practice patterns rather than causation.

## Highlights

• The use of tMCS increased with the largest increases noted for Impella and ECMO.
• Use of ECMO coincided with higher 30-day mortality compared with IABP.
• Overall hospitalization cost was highest for ECMO and lowest for IABP.
• These findings likely reflect increasing disease severity and evolving practice.

## Central Illustration

**Figure:**
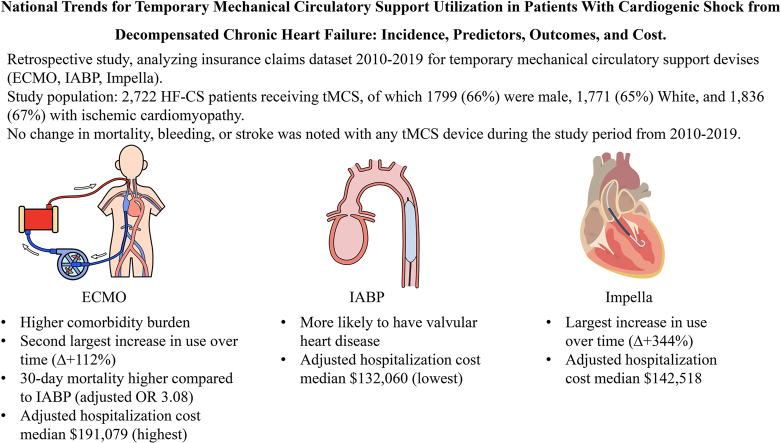

## Introduction

The last decade has seen rapid technological advances in temporary mechanical circulatory support (tMCS) devices. These devices have primarily been studied in acute myocardial infarction leading to cardiogenic shock (AMI-CS) or high-risk percutaneous coronary intervention (PCI).^1^, ^2^, ^3^, ^4^, ^5^ Apart from AMI-CS, patients with advanced heart failure (HF) often present in an acute decompensated state and represent approximately 30% of all patients with cardiogenic shock.^6^ tMCS utilization in patients with cardiogenic shock due to decompensated chronic heart failure (HF-CS) is poorly understood. Most reports thus far have been limited to single-center experiences or have focused on patients receiving cardiac transplants or durable surgical left ventricular assist devices.^7^, ^8^, ^9^ Accordingly, there remains a knowledge gap regarding how tMCS devices are utilized in all patients with HF-CS, with limited real-world data describing outcomes for these patients.

We seek to address this knowledge gap by describing the contemporary use of tMCS devices, namely Impella (Abiomed), intra-aortic balloon pump (IABP), or extracorporeal membrane oxygenation (ECMO) for patients with HF-CS in the absence of AMI-CS, high-risk PCI, or postcardiotomy shock in a national dataset. We also identify predictors for the type of device used and associated outcomes, including 30-day mortality, bleeding, and stroke. Finally, we assess hospitalization costs associated with tMCS devices in this cohort and examine the correlation between costs and outcomes.

## Methods

### Data sources

Optum Clinformatics Data Mart is a deidentified database of administrative health claims for over 80 million commercially insured beneficiaries enrolled in private and Medicare Advantage health plans.^10^ The database includes inpatient and outpatient claims for enrolled individuals in all 50 states. It also includes data on inpatient procedures and related expenditures for enrolled individuals. Since patient-level data are deidentified, the University of Michigan Institutional Review Board determined the study to be exempt.

### Study design, setting, and population

This is a retrospective cohort study of individuals enrolled in the Optum Clinformatics Data Mart from January 1, 2010, to December 31, 2019. We included all individuals ≥18 years, with at least 1 outpatient encounter or 1 inpatient encounter with International Classification of Diseases (ICD), 9th or 10th Revision codes for HF or cardiogenic shock who received a form of tMCS device (ie, Impella, IABP, or ECMO). These codes have been previously validated and used in literature and are endorsed by the Centers for Medicare and Medicaid for use with claims data.^11^, ^12^, ^13^, ^14^ To identify patients who received tMCS devices, current procedural terminology (CPT) codes from inpatient claims data were used (Supplemental Table S1).

We excluded patients with AMI or PCI during the same hospitalization to exclude patients representing cardiogenic shock secondary to an acute coronary syndrome. Existing studies have reported an increase in use of tMCS for high-risk PCI.^15^ Accordingly, to solely capture patients who received tMCS for HF-CS, we excluded patients undergoing PCI. We also excluded patients who underwent cardiothoracic surgery for valvular replacement or coronary artery bypass grafting to exclude patients with postcardiotomy cardiogenic shock. Since patients may receive more than 1 tMCS device during hospitalization, all patients who received ECMO in addition to another device were classified in the ECMO group. Patients who received both an IABP and Impella were excluded, and only those who received either an IABP or Impella were included in the study. We required all individuals to enroll in a medical plan for at least 6 months before their index date to capture their comorbidity burden accurately.

We further identified patients with ischemic cardiomyopathy using validated ICD-9/10 codes (Supplemental Table S1) within 1 year before index hospitalization of device insertion.^12^ All other patients without codes for ischemic cardiomyopathy were classified as having nonischemic cardiomyopathy. The index date for the study cohort was the date of the first CPT code for any tMCS device within the study period.

### Variables

#### Predictors of type of tMCS device

Variables of interest included patient demographic characteristics and comorbidities. Demographic characteristics included age, sex, race, health plan coverage, and geographical region at the service date of the device insertion. Race was determined using information obtained by Optum Insight from public records (eg, driver’s license data), the first and last name of the beneficiary, and the census block of residence. Comorbidities were identified using previously validated ICD-9 and ICD-10 codes in any position in the 6 months before the index date (Supplemental Table S1).^16^ To examine geographic variation in utilization, states were grouped into census divisions and regions.^17^

#### Outcomes with tMCS

We examined the association between device type and 30-day all-cause mortality. Additional outcomes assessed included clinically significant bleeding needing transfusions and stroke during index hospitalization using validated ICD-9/10 codes (Supplemental Table S1).^18^^,^^19^ These outcomes were assessed after the date of the CPT code for a tMCS device. We also assessed the total cost incurred during the hospitalization that the patient received a tMCS device.

### Statistical analysis

Patient characteristics were compared between patients who received an IABP, Impella, or ECMO. We used the χ^2^ test for categorical variables, and for normally distributed continuous variables, we used the analysis of variance test. To examine trends in the use of tMCS devices, we performed linear regression analysis for each device. Next, to identify factors associated with receiving a specific type of tMCS device, we used a multinomial logistic regression model fitted with patient demographic characteristics such as age, sex, race, region, and clinical characteristics such as drug/alcohol abuse, diabetes, hypertension, cardiac arrest, atrial fibrillation, acute kidney injury (AKI), ventricular tachycardia/fibrillation, stroke, valvular disease, peripheral vascular disease, chronic obstructive pulmonary disease, chronic kidney disease (CKD), coagulopathy, obesity, cachexia, and depression. These variables were selected based on clinical rationale. We used IABP as a reference category in this multinomial logistic regression. Predictors for the tMCS device type used were assessed separately for patients with ischemic and nonischemic cardiomyopathy. To assess if our results vary by ICD codes used, we repeated the analyses above for the cohort prior to October 2015 (ie, ICD-9 cohort) and after October 2015 (ie, ICD-10 cohort). Results were similar for both subcohorts and are provided in Supplemental Table S2.

To examine the association between the type of tMCS device used and outcomes (30-day mortality, stroke), we created a multivariable logistic regression with the model adjusted for the patient covariates described above. For the cost analyses, we summed all the payments (including provider and hospital payments) available in the claims to gather the total cost of hospitalization at the patient level. Total costs were inflation-adjusted to 2019 dollars using a proprietary cost factor multiplier table provided by Optum Insight. The cost factor multipliers are patient setting-specific multipliers. We standardized observed total hospitalization cost after adjusting for patient demographic characteristics, comorbidities, year of the service, type of devices, insurance type, health maintenance organization plan, postimplant length of stay, and hospital level characteristics (number of beds, region).

To examine the association between costs and associated outcomes, we rank-ordered price-standardized total cost into 4 quartiles. We used generalized linear models (GLM) using PROC GENMOD in SAS (SAS Institute) using a log link and Poisson distribution to evaluate the association between adverse events and price-standardized payment quartiles. GLM models were adjusted for the patient demographic characteristics, comorbidities, and clinical status.

All analyses were conducted using SAS version 9.4. PROC LOGISTIC with generalized logit model (glogit link) was used to fit the type of the device’s model PROC GENMOD was used for the price-standardized model, and PROC LOGISTIC with logit link for fitting models for the adverse events like 30-day mortality, stroke, and bleeding.

## Results

We identified 2722 patients who received tMCS devices of interest, ie, IABP, Impella, or ECMO, with previously diagnosed HF or cardiogenic shock without AMI, cardiac surgery, or PCI. This included 1836 (67%) adults with ischemic cardiomyopathy. Patients receiving ECMO were more likely to be younger than IABP and Impella patients and more likely to have nonischemic cardiomyopathy. Table 1 shows the baseline characteristics of the cohort stratified by the type of tMCS device used.

**Table 1:** Patient baseline characteristics stratified by type of temporary mechanical circulatory support received.

Variables	IABP (n = 1630)	Impella (n = 508)	ECMO (n = 584)	All (N = 2722)	*P* value
Male sex	1067 (65.4%)	382 (75.2%)	350 (59.9%)	1799 (66.1%)	.02
Insurance					<.01
Commercial	771 (47.3%)	182 (35.8%)	387 (66.3%)	1340 (49.2%)	
Medicare Advantage	859 (52.7%)	326 (64.2%)	197 (33.7%)	1382 (50.8%)	
HMO	264 (16.2%)	56 (11.0%)	69 (11.8%)	389 (14.3%)	.10
Race					.52
White	1057 (64.8%)	335 (65.9%)	379 (64.9%)	1771 (65.1%)	
Black	245 (15.0%)	57 (11.2%)	75 (12.8%)	377 (13.8%)	
Asian	39 (2.4%)	12 (2.4%)	18 (3.1%)	69 (2.5%)	
Hispanic ethnicity	105 (6.4%)	32 (6.3%)	51 (8.7%)	188 (6.9%)	
Age, y					<.01
18-44	174 (10.7%)	50 (9.8%)	158 (27.0%)	382 (14.0%)	
45-64	685 (42.0%)	176 (34.6%)	285 (48.8%)	1146 (42.1%)	
≥65	771 (47.3%)	282 (55.5%)	141 (24.1%)	1194 (43.9%)	
Region					<.01
Midwest	463 (28.4%)	101 (19.9%)	125 (21.4%)	689 (25.3%)	
Northeast	192 (11.8%)	55 (10.8%)	99 (16.9%)	346 (12.7%)	
South	768 (47.1%)	268 (52.7%)	277 (47.4%)	1313 (48.2%)	
West	207 (12.7%)	84 (16.5%)	83 (14.2%)	374 (13.7%)	
Ischemic cardiomyopathy	1146 (70.3%)	409 (80.5%)	281 (48.1%)	1836 (67.4%)	<.01
Cardiac arrest	57 (3.5%)	30 (2.0%)	75 (12.8%)	162 (5.9%)	<.01
Atrial fibrillation	454 (27.8%)	215 (42.3%)	174 (29.8%)	843 (31.0%)	.76
Ventricular arrhythmias	289 (17.7%)	146 (28.7%)	119 (20.4%)	554 (20.3%)	<.01
Stroke	88 (5.4%)	12 (2.4%)	36 (3.1%)	136 (5%)	.22
Diabetes mellitus	669 (41.0%)	241 (47.4%)	177 (13.2%)	1087 (40.0%)	.05
Hypertension	1302 (79.9%)	414 (81.5%)	411 (70.4%)	2127 (78.1%)	.01
Valvular disease	1281 (78.6%)	319 (62.8%)	347 (59.4%)	1947 (71.5%)	<.01
Pulmonary vascular disorder	707 (43.4%)	170 (33.5%)	266 (45.5%)	1143 (42.0%)	.06
Peripheral vascular disease	673 (41.3%)	285 (56.1%)	229 (39.2%)	1187 (43.6%)	<.01
Paralysis	29 (1.8%)	11 (2.2%)	25 (4.3%)	65 (2.4%)	.30
Chronic pulmonary disease	696 (42.7%)	161 (31.7%)	240 (41.1%)	1097 (40.3%)	.07
Hypothyroidism	322 (19.7%)	116 (22.8%)	106 (18.1%)	544 (20.0%)	.76
Acute kidney injury	734 (45.0%)	256 (50.4%)	386 (66.1%)	1376 (50.5%)	<.01
Chronic renal failure	704 (43.2%)	237 (46.6%)	235 (40.2%)	1176 (43.2%)	.13
Chronic liver disease	310 (19.0%)	122 (24.0%)	226 (38.7%)	658 (24.2%)	<.01
Malignancy	175 (10.7%)	57 (11.2%)	68 (11.6%)	300 (11.0%)	.31
Coagulopathy	444 (27.2%)	131 (25.8%)	300 (51.4%)	875 (32.1%)	<.01
Obesity	356 (21.8%)	119 (23.4%)	139 (23.8%)	614 (22.5%)	.98
Cachexia	250 (15.3%)	74 (14.6%)	174 (29.8%)	498 (18.3%)	<.01
Fluid and electrolyte disorder	917 (56.2%)	290 (57.1%)	421 (72.1%)	1628 (59.8%)	<.01
Blood loss anemia	64 (3.9%)	15 (2.9%)	42 (7.2%)	121 (4.4%)	.02
Alcohol use disorder	92 (5.6%)	35 (6.9%)	37 (6.3%)	164 (6.0%)	.09
Substance use disorder	43 (2.6%)	21 (4.1%)	38 (6.5%)	102 (3.7%)	<.01
Psychosis	31 (1.9%)	8 (1.5%)	12 (2.0%)	51 (1.9%)	.42
Depression	273 (16.7%)	85 (16.7%)	127 (21.7%)	485 (17.8%)	<.01

Data are presented as n (%) for categorical data. *P* value < .05 is defined as statistically significant difference between comparison groups.

ECMO, extracorporeal membrane oxygenation; HMO, health maintenance organization; IABP, intra-aortic balloon pump.

### Temporal trend and predictors for tMCS device selection

The Central Illustration shows temporal trends in tMCS device selection from 2010 to 2019. Overall, the number of HF-CS patients receiving tMCS increased. The largest change in device use when comparing 2019 to 2010 usage was found for Impella (+344.4%), followed by ECMO (+112.4%).

**Central Illustration:**
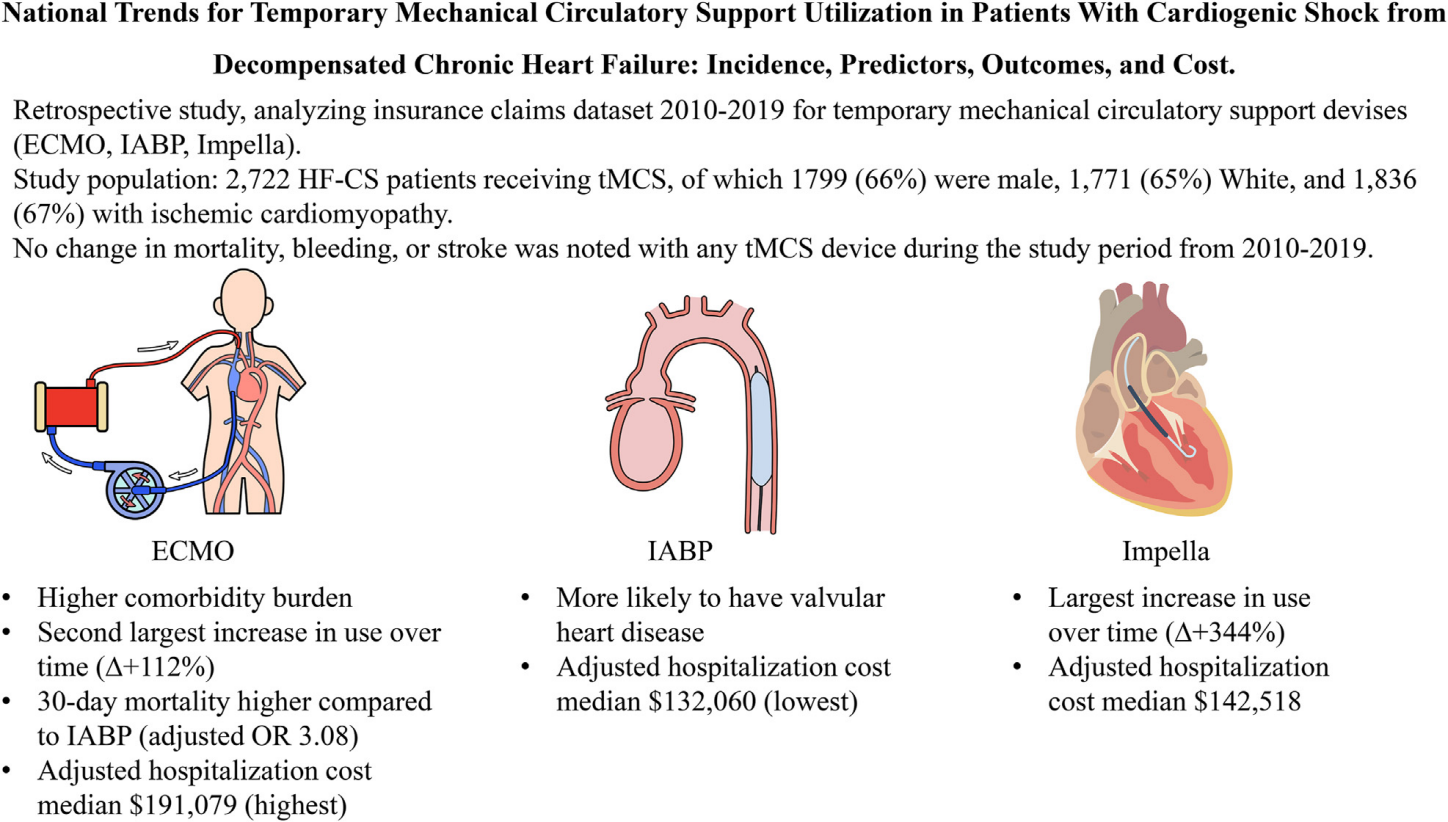
Trends, predictors, outcomes, and cost associated with temporary mechanical circulatory support device use in cardiogenic shock secondary to decompensated chronic heart failure.

### Device-use patterns in ischemic cardiomyopathy

Table 2 shows predictors for device type in patients stratified by ischemic and nonischemic cardiomyopathy. Patients with valvular disease were less likely to receive ECMO (odds ratio [OR], 0.48; 95% CI, 0.36-0.65) or Impella (OR, 0.51; 95% CI, 0.39-0.66) than IABP. Patients with CKD were also less likely to receive ECMO (OR, 0.66; 95% CI, 0.45-0.98) or Impella (OR, 0.53; 95% CI, 0.37-0.77) compared with IABP. Patients with AKI were more likely to receive ECMO (OR, 1.70; 95% CI, 1.24-2.33) or Impella (OR, 1.34; 95% CI, 1.02-1.75) compared with IABP. Patients with peripheral arterial disease were also more likely to receive an Impella than IABP (OR, 1.30; 95% CI, 1.00-1.69), and patients with coagulopathy were more likely to receive ECMO than IABP (OR, 2.06; 95% CI, 1.54-2.77). Race and sex were not significant predictors of device type.

**Table 2:** Predictors of temporary mechanical circulatory support device selection in ischemic and nonischemic cardiomyopathy patients.

Variable	Ischemic cardiomyopathy^a^ aOR (95% CI)		Nonischemic cardiomyopathy^a^ aOR (95% CI)	
	ECMO	Impella	ECMO	Impella
Female sex	1.31 (0.98-1.74)	0.95 (0.73-1.25)	1.28 (0.93-1.77)	1.00 (0.64-1.58)
Race (White)	0.86 (0.59-1.24)	0.94 (0.68-1.30)	1.06 (0.65-1.72)	0.95 (0.51-1.76)
Commercial insurance	0.93 (0.69-1.25)	0.63 (0.48-0.82)	1.62 (1.09-2.40)	1.09 (0.64-1.85)
Hispanic	0.86 (0.49-1.52)	0.76 (0.45-1.27)	2.32 (1.11-4.81)	0.83 (0.28-2.49)
Diabetes mellitus	0.84 (0.64-1.11)	1.09 (0.86-1.39)	0.66 (0.45-0.99)	1.49 (0.89-2.47)
Hypertension	0.89 (0.61-1.30)	1.08 (0.76-1.54)	0.71 (0.50-1.01)	0.55 (0.34-0.89)
Cardiac arrest	3.21 (1.98-5.19)	1.43 (0.84-2.41)	1.67 (0.89-3.12)	0.98 (0.43-2.23)
Atrial fibrillation	1.10 (0.80-1.50)	1.36 (1.05-1.77)	0.95 (0.61-1.49)	0.55 (0.31-0.99)
Acute kidney injury	1.70 (1.24-2.33)	1.34 (1.02-1.75)	2.54 (1.69-3.82)	3.10 (1.76-5.45)
Ventricular tachycardia	0.85 (0.59-1.20)	1.01 (0.81-1.47)	0.97 (0.59-1.60)	1.75 (0.96-3.19)
Valvular heart disease	0.48 (0.36-0.65)	0.51 (0.39-0.66)	0.29 (0.21-0.41)	0.36 (0.23-0.57)
Stroke	1.05 (0.60-1.84)	0.93 (0.49-1.77)	1.48 (0.72-3.02)	1.29 (0.48-3.51)
Peripheral arterial disease	0.91 (0.68-1.23)	1.30 (1.00-1.69)	1.19 (0.81-1.74)	2.53 (1.53-4.16)
COPD	1.03 (0.79-1.35)	0.62 (0.49-0.79)	1.77 (1.25-2.51)	0.96 (0.58-1.59)
Chronic kidney disease	0.66 (0.45-0.98)	0.53 (0.37-0.77)	0.67 (0.43-1.05)	0.44 (0.23-0.85)
Coagulopathy	2.06 (1.54-2.77)	0.76 (0.57-1.01)	2.45 (1.73-3.47)	1.62 (1.00-2.64)
Obesity	1.18 (0.86-1.61)	0.97 (0.73-1.27)	0.74 (0.50-1.11)	0.64 (0.37-1.11)
Cachexia	1.27 (0.93-1.75)	0.79 (0.56-1.09)	1.54 (1.04-2.29)	0.84 (0.47-1.50)
Blood loss anemia	1.38 (0.84-2.29)	0.54 (0.28-1.04)	2.21 (0.92-5.31)	1.52 (0.43-5.45)
Alcohol/substance use disorder	0.82 (0.51-1.33)	1.28 (0.84-1.95)	1.47 (0.91-2.36)	1.26 (0.66-2.38)

aOR, adjusted odds ratio; COPD, chronic obstructive pulmonary disease.

a Intra-aortic balloon pump as reference.

### Device-use patterns in nonischemic cardiomyopathy

Patients with valvular disease were similarly less likely to receive ECMO (OR, 0.29; 95% CI, 0.21-0.41) or Impella (OR, 0.36; 95% CI, 0.23-0.57) than IABP. Patients with CKD were also less likely to receive Impella (OR, 0.44; 95% CI, 0.23-0.85) compared with IABP. Patients with AKI were more likely to receive ECMO (OR, 2.54; 95% CI, 1.69-3.82) or Impella (OR, 3.10; 95% CI, 1.76-5.45) compared with IABP. Similarly, patients with peripheral arterial disease were more likely to receive an Impella than IABP (OR, 2.53; 95% CI, 1.53-4.16). Patients with coagulopathy were also more likely to receive ECMO than IABP (OR, 2.45; 95% CI, 1.73-3.47). Race and sex were not significant predictors of device type.

### Association between tMCS device type and outcomes

**Supplementary Figure S1B:**
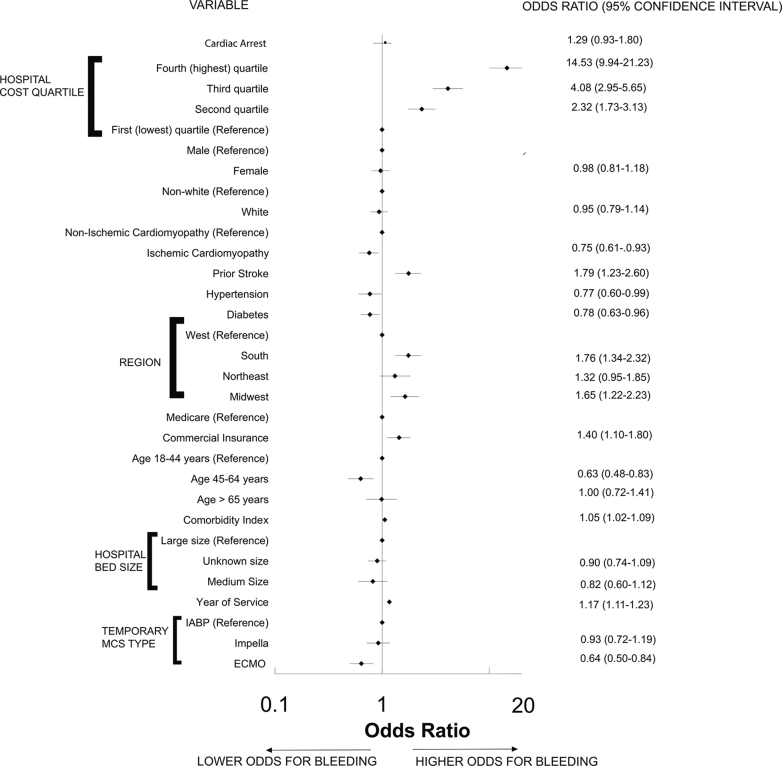


Observed 30-day mortality rates were highest in patients with ECMO (26%, n = 154), followed by Impella (20%, n = 101) and IABP (17%, n = 277). After multivariable adjustment, there was no difference in 30-day mortality between Impella and IABP (adjusted OR [aOR], 1.24; 95% CI, 0.93-1.66). However, odds for 30-day mortality were significantly higher with ECMO compared with IABP (aOR, 3.08; 95% CI, 2.22- 4.27). Age, insurance type, and cardiac arrest were the only patient characteristics associated with 30-day mortality (Supplemental Figure S1A). Compared with adults under 45 years, we noted increasing odds for mortality for patients between 45 and 64 years (aOR, 2.34; 95% CI, 1.59-3.43) and for patients over 65 years (aOR, 3.05; 95% CI, 1.94-4.80). Patients with cardiac arrest had higher 30-day mortality (aOR, 1.61; 95% CI, 1.10-2.35).

We observed that in-hospital bleeding rates after implantation of tMCS were highest in patients with ECMO (42%, n = 246), followed by Impella (28%, n = 181) and IABP (22%, n = 354). After multivariable adjustment, compared with IABP, the odds for bleeding were lower with ECMO (aOR, 0.64; 95% CI, 0.50-0.83). However, compared with IABP, the odds for bleeding were not significantly different with Impella (aOR, 0.92; 95% CI, 0.72-1.18). All other patient and hospital characteristics associated with in-hospital bleeding are shown in Supplemental Figure S1B. There were no race or sex-based differences in risk for in-hospital bleeding. Increasing comorbidity burden was associated with increased risk for in-hospital bleeding (aOR, 1.05; 95% CI, 1.02-1.09 per unit increase in Charleston comorbidity index). History of previous stroke also correlated with in-hospital bleeding risk (aOR, 1.79; 95% CI, 1.23-2.60).

Rates of in-hospital stroke after implantation of tMCS were highest for ECMO (6%, n = 36), followed by Impella (5.4%, n = 88) and IABP (2.4%, n = 12). Following multivariable adjustment, the odds for stroke risk with ECMO (aOR, 0.81; 95% CI, 0.58-1.14) and Impella (aOR, 1.09; 95% CI, 0.79-1.49) compared with IABP were not statistically significant. Supplemental Figure S1C shows predictors for stroke risk in patients receiving tMCS. Patient factors associated with stroke risk included race, with lower odds for stroke in White patients compared with non-White (aOR, 0.74; 95% CI, 0.58-0.94). Cardiac arrest was also associated with a higher risk for stroke (aOR, 2.68; 95% CI, 1.85-3.89). The strongest risk factor for stroke was a previous history of stroke (aOR, 28.47; 95% CI, 8.52-43.78).

**Figure 1:**
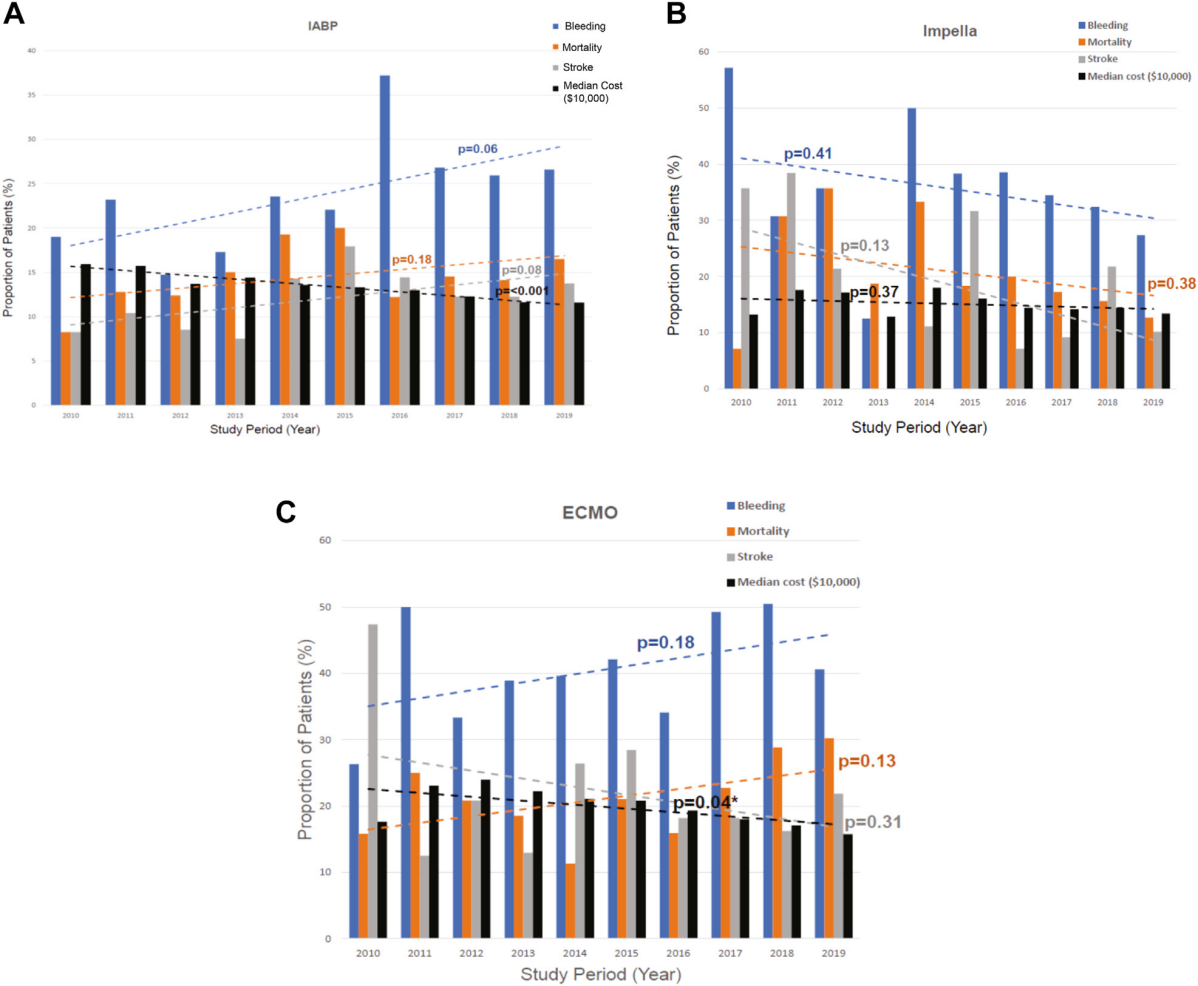
**Temporal trend in risk of 30-day mortality, bleeding, stroke, and cost stratified by type of temporary mechanical circulatory support used from 2010-2019.** (**A**) IABP (intra-aortic balloon pump), (**B**) Impella, (**C**) extracorporeal membrane oxygenation (ECMO).

Over the entire study period, we noted no change in 30-day mortality, bleeding, and hospitalization rates with any of the devices (Figure 1).

### Cost and correlation with outcomes

For patients receiving an IABP, the adjusted median cost of hospitalization was lowest (median, $132,060 [IQR, $113,794-$160,244]; mean, $143,726 ± $42,950) followed by Impella (median, $142,518 [IQR, $126,845-$179,938]; mean, $163,718 ± $53,919). The adjusted cost of hospitalization was highest among patients receiving ECMO support (median, $191,079 [IQR, $165,760-$239,373]; mean, $210,383 ± $66,028). During the study period, we noted a decrease in hospitalization costs with IABP (*P* < .01) and ECMO (*P* = .04) but not with Impella (*P* = .37) over time (Figure 1).

We divided sites into 4 quartiles based on hospitalization cost incurred to examine the association between cost and outcomes with tMCS devices. The observed hospitalization cost difference was 1348% between the highest ($343,056 ± $48,292) and lowest ($23,693 ± $24,055) quartiles. After adjustment for patient and hospital characteristics, this difference dropped to 126% between the highest ($247,622 ± $51,048) and lowest ($109,627 ± $8884) quartiles. After adjusting for patient factors, we observed a direct and linear association between price standardized cost-quartile and stroke and bleeding odds (Supplemental Figure S1B, C). However, after adjusting for patient factors, adjusted odds for 30-day mortality were low only in the highest quartile for standardized cost compared with the lowest quartile.

## Discussion

Temporary mechanical circulatory support devices have been predominantly studied in the context of cardiogenic shock in AMI or high-risk PCI. However, recent literature suggests that the epidemiology of cardiogenic shock is changing with an increase in the proportion of patients with cardiogenic shock secondary to decompensated chronic HF.^20^ Recent changes in the cardiac transplant allocation system have also increased the use of tMCS in transplant candidates. Existing studies assessing the use of tMCS for HF have focused on specific patients receiving these devices.^7^ Accordingly, there is a need for more data on the use of tMCS in HF patients, and our study addresses this knowledge gap by describing a nationwide evolution in tMCS device use in patients with HF-CS.

Overall, the use of tMCS devices is on the rise. However, studies in Europe suggest that there has been a decline in IABP use since the IABP-SHOCK II clinical trial.^4^^,^^21^ In contrast, our study shows an increase in use of all tMCS devices, with the largest increase noted for Impella followed by ECMO. Similar to our study, others have previously described increased Impella and ECMO use in patients with post-AMI and postcardiotomy cardiogenic shock.^22^^,^^23^ However, despite the increase in tMCS device use, over the entire duration of our study from 2010-2019, we noted no differences in bleeding, 30-day mortality, or hospitalization rate for each device type suggesting that an increase in device use has not translated to improved outcomes.

We also describe contemporary, real-world outcomes associated with tMCS in HF-CS patients. We noted higher 30-day mortality in patients receiving ECMO compared with IABP, with no difference in 30-day mortality between Impella and IABP. The stroke rates were no different between ECMO and Impella compared with IABP. Notably, there were no race or sex differences seen for bleeding or mortality. The correlation between ECMO and worse outcomes likely reflects increasing disease severity and evolving practice patterns rather than causal inference. This is highlighted in our models assessing predictors for ECMO use that included higher acuity patients such as those with cardiac arrest, AKI, and coagulopathy. Similarly, Impella use was also associated with signs of increased illness severity, such as AKI. Compared with ECMO, evaluation of predictors for Impella use showed regional variation in the use of this device with decreased use in the Midwest and Northeastern regions compared to the West. This suggests variability in the type of device used based on operator preferences and hospital practices, similar to other data describing site-level variation in Impella use for patients undergoing high-risk PCI.^23^ These results underscore the need for higher-quality prospective evidence to inform clinical practice using tMCS in HF-CS.

We noted a significant difference in hospitalization costs with the type of tMCS device used and a direct relationship between price-adjusted hospitalization cost and device-associated complications of stroke and bleeding. Still, this relationship was not noted for 30-day mortality. We also noted a high-cost burden associated with using tMCS devices with significant differences in cost associated with Impella and ECMO compared with IABP. While we noted a direct correlation between cost and complications, hospitalization cost once again did not correlate with 30-day mortality. Our study also identified that specific patient characteristics such as older age and previous history of stroke were associated with higher mortality and higher risk for complications. In addition, we noted no change in outcomes with tMCS over time despite an increase in their use. While increasing costs did not necessarily lead to better survival, it is reassuring to see similar 30-day mortality between groups, as increasing costs may be a marker of higher disease severity in such patients.

### Limitations

The findings of our study should be interpreted in the light of several limitations. Inherent to all retrospective observational studies, we cannot rule out residual confounding due to unmeasured factors such as prior HF medical therapy, patient-level hemodynamic data, use of vasoactive medications, or noncardiac organ support devices. Our findings cannot assume causality. Using a national claims-based dataset, we describe real-world practice patterns regarding the use of tMCS in HF patients with costs and associated outcomes. Since we used an administrative dataset, death, bleeding, and stroke outcomes are derived from administrative codes with the potential for misclassification. We, however, examined the presence of these events, specifically after the date of a patient receiving a tMCS device. We also could not account for the type of Impella device or ECMO system used. We were only able to adjust for limited site-level variables and lacked granular data on additional site-level characteristics such as hospital volumes for device volumes. Lastly, our cost analysis is limited to hospitalization costs, and long-term costs from a societal perspective may differ in such patients.

## Conclusion

In a real-world cohort of patients with HF-CS, the use of tMCS has increased with the largest increases noted for Impella and ECMO over the last decade. Patients receiving ECMO have a higher comorbidity burden, and use of ECMO is associated with a higher rate of 30-day mortality, compared with IABP, with no difference in outcomes between Impella and IABP. tMCS device use was associated with high costs during hospitalization that correlated with complications of stroke and bleeding but not with 30-day mortality.

## Supplementary material

Supplementary Figure S1A

Supplementary Figure S1C
