# Supplementary figures and images for: National Trends for Temporary Mechanical Circulatory Support Utilization in Patients With Cardiogenic Shock From Decompensated Chronic Heart Failure: Incidence, Predictors, Outcomes, and Cost
Source: J Soc Cardiovasc Angiogr Interv. 2023 Dec 4;2(6Part B):101177. doi: 10.1016/j.jscai.2023.101177 (PMC11307713; doi:10.1016/j.jscai.2023.101177)

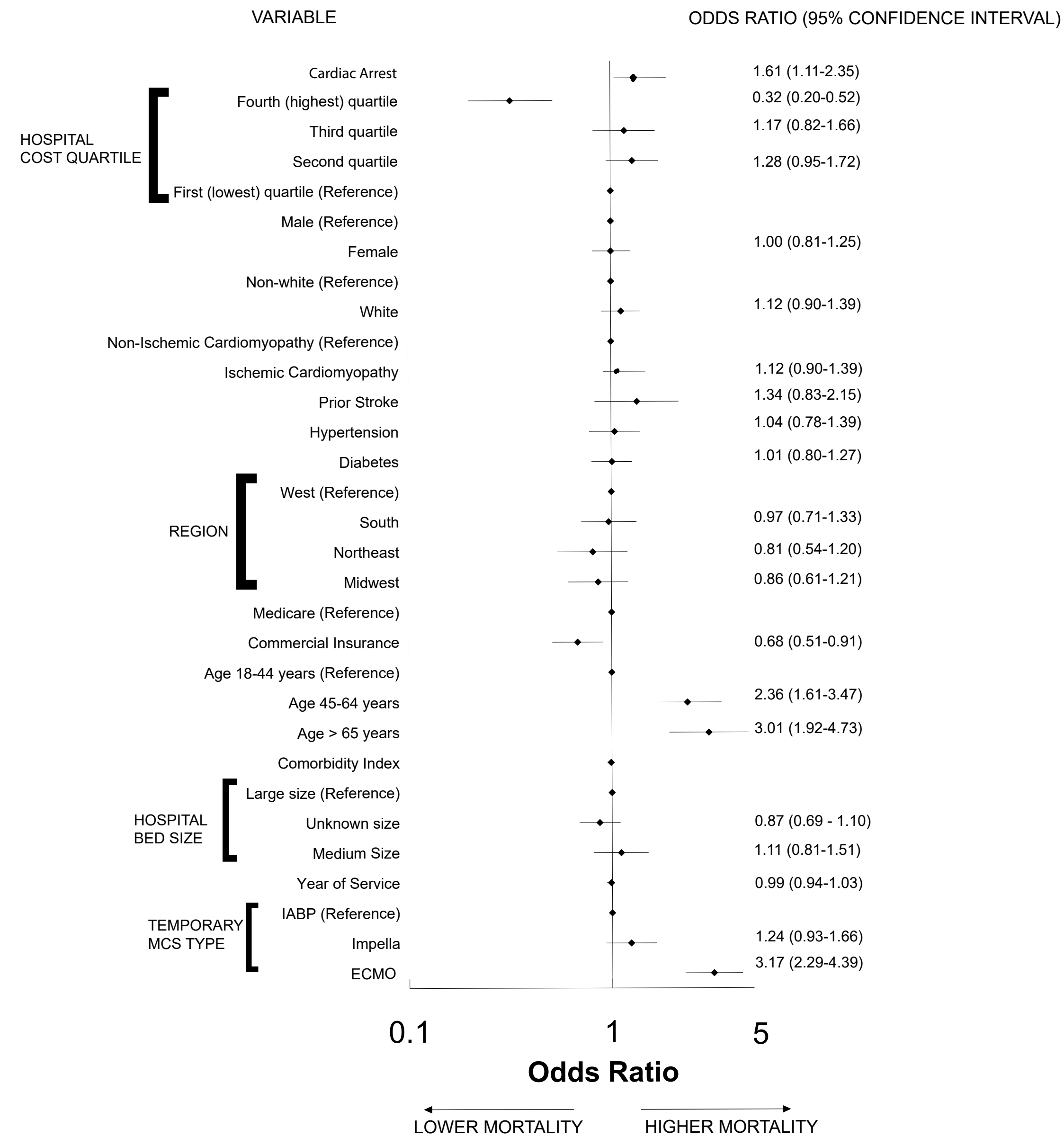

Supplement: Supplementary Figure S1A [file mmc1.pdf]

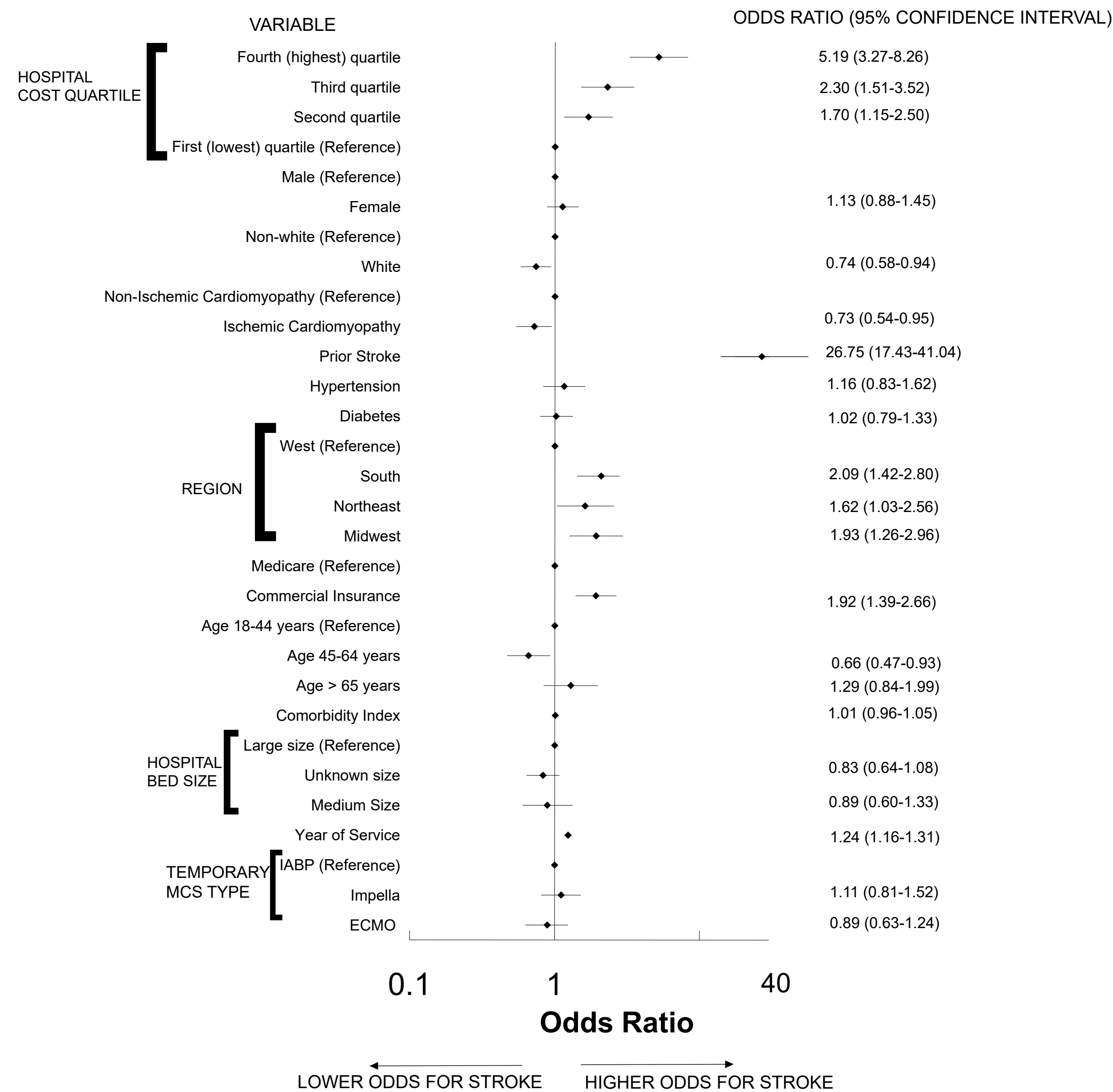

Supplement: Supplementary Figure S1C [file mmc2.pdf]
