# Supplementary material for: National Trends for Temporary Mechanical Circulatory Support Utilization in Patients With Cardiogenic Shock From Decompensated Chronic Heart Failure: Incidence, Predictors, Outcomes, and Cost
Source: J Soc Cardiovasc Angiogr Interv. 2023 Dec 4;2(6Part B):101177. doi: 10.1016/j.jscai.2023.101177 (PMC11307713; doi:10.1016/j.jscai.2023.101177)
Supplement: Supplemental Table S1 [file mmc3.docx]

Supplemental Table 1: Administrative codes used in the study

| Heart Failure | |
| --- | --- |
| ICD 9 | 398.91, 402.01, 402.11, 402.91, 404.01, 404.03, 404.11, 404.13, 404.91, 404.93, 428.0, 428.1, 428.20, 428.21, 428.22, 428.23, 428.30, 428.31, 428.32, 428.33, 428.40, 428.41, 428.42, 428.43, 428.9 |
| ICD 10 | I09.81, I11.0, I13.0, I13.2, I50.1, I50.20, I50.21, I50.22, I50.23, I50.30, I50.31, I50.32, I50.33, I50.40, I50.41, I50.42, I50.43, I50.810, I50.811, I50.812, I50.813, I50.814, I50.82, I50.83, I50.84, I50.89, I50.9 |
| Cardiogenic shock | |
| ICD 9 | 785.51 |
| ICD 10 | R57.0 |
| AMI | |
| ICD 9 | 410.01, 410.11, 410.21, 410.31, 410.41, 410.51, 410.61, 410.71, 410.81, 410.91 |
| ICD 10 | I21.01, I21.02, I21.09, I21.11, I21.19, I21.21, I21.29, I21.3, I21.4, I21.9, I21.A1, I21.A9, I22.0, I22.1, I22.2, I22.8, I22.9 |
| PCI | |
| ICD 9 | 00.66, 17.55, 36.01, 36.02, 36.05, 36.06, 36.07, 36.09 |
| ICD 10 | 0270046, 027004Z, 02700D6, 02700DZ, 02700T6, 02700TZ, 02700Z6, 02700ZZ, 0270346, 027034Z, 02703D6, 02703DZ, 02703T6, 02703TZ, 02703Z6, 02703ZZ, 0270446, 027044Z, 02704D6, 02704DZ, 02704T6, 02704TZ, 02704Z6, 02704ZZ, 0271046, 027104Z, 02710D6, 02710DZ, 02710T6, 02710TZ, 02710Z6, 02710ZZ, 0271346, 027134Z, 02713D6, 02713DZ, 02713T6, 02713TZ, 02713Z6, 02713ZZ, 0271446, 027144Z, 02714D6, 02714DZ, 02714T6, 02714TZ, 02714Z6, 02714ZZ, 0272046, 027204Z, 02720D6, 02720DZ, 02720T6, 02720TZ, 02720Z6, 02720ZZ, 0272346, 027234Z, 02723D6, 02723DZ, 02723T6, 02723TZ, 02723Z6, 02723ZZ, 0272446, 027244Z, 02724D6, 02724DZ, 02724T6, 02724TZ, 02724Z6, 02724ZZ, 0273046, 027304Z, 02730D6, 02730DZ, 02730T6, 02730TZ, 02730Z6, 02730ZZ, 0273346, 027334Z, 02733D6, 02733DZ, 02733T6, 02733TZ, 02733Z6, 02733ZZ, 0273446, 027344Z, 02734D6, 02734DZ, 02734T6, 02734TZ, 02734Z6, 02734ZZ, 02C03ZZ, 02C04ZZ, 02C13ZZ, 02C14ZZ, 02C23ZZ, 02C24ZZ, 02C33ZZ, 02C34ZZ, X2C0361, X2C1361 |
| Post cardiotomy | |
| ICD 9 | 35.10–35.14, 35.20–35.28, 36.10-36.17, 36.19 |
| ICD 10 | 02QF0ZZ, 02QG0ZZ, 02QH0ZZ, 02QJ0ZZ, 02RF07Z, 02RF08Z, 02RF0JZ, 02RF0KZ, 02RF47Z, 02RF48Z, 02RF4JZ , 02RF4KZ, 02RG07Z, 02RG08Z, 02RG0JZ,  02RG0KZ,  02RG47Z,  02RG48Z,  02RG4JZ,  02RG4KZ, 02RH07Z,  02RH08Z, 02RH0JZ, 02RH0KZ,  02RH47Z,  02RH48Z, 02RH4JZ, 02RH4KZ,  02RJ07Z,  02RJ08Z,  02RJ0JZ, 02RJ0KZ, 02RJ47Z, 02RJ48Z, 02RJ4JZ,  02RJ4KZ, 0210093, 0210098, 0210099, 021009C, 021009F, 021009W, 02100A3, 02100A8, 02100A9, 02100AC, 02100AF, 02100AW, 021200J3, 02100J8, 02100J9, 02100JC, 02100JF, 02100JW, 02100K3, 02100K8, 02100K9, 02100KC, 02100KF, 02100KW, 02100Z3, 02100Z8, 02100Z9, 02100ZC, 02100ZF, 0211093, 0211098, 0211099, 021109C, 021109F, 021109W, 02110A3, 02110A8, 02110A9, 02110AC, 02110AF, 02110AW, 02110J3, 02110J8, 02110J9, 02110JC, 02110JF, 02110JW, 02110K3, 02110K8, 02110K9, 02110KC, 02110KF, 02110KW, 02110Z3, 02110Z8, 02110Z9, 02110ZC, 02110ZF, 0212093, 0212098, 0212099, 021209C, 021209F, 021209W, 02120A3, 0212OA8, 02120A9, 02120AC, 02120AF, 02120AW, 02120J3, 02120J8, 02120J9, 02120JC, 02120JF, 02120JW, 02120K3, 02120K8, 02120K9, 02120KC, 02120KF, 02120KW, 02120Z3, 02120Z8, 02120Z9, 02120ZC, 02120ZF, 0213093, 0213098, 0213099, 021309C, 021309F, 021309W, 02130A3, 02130A8, 02130A9, 02130AC, 02130AF, 02130AW, 02130J3, 02130J8, 02130J9, 02130JC, 02130JF, 02130JW, 02130K3, 02130K8, 02130K9, 02130KC, 02130KF, 02130KW, 02130Z3, 02130Z8, 02130Z9, 02130ZC, 02130ZF |
| Intra-Aortic Balloon Pump | |
| ICD 9 | 37.61 |
| ICD 10 | 5A02110, 5A02210 |
| Impella | |
| ICD 9 | 37.68 |
| ICD 10 | 5A0211D, 5A0221D |
| Extra Corporeal Membrane Oxygenation (ECMO) | |
| ICD 9 | 39.65 |
| ICD 10 | 5A1522G |
| Stroke | |
| ICD 9 | 433.01, 433.01, 433.21, 433.31, 433.81, 433.91, 434.01, 434.11, 434.91, 430, 431, 432.0, 432.1, 432.9, 435.0, 435.1, 435.2, 435.3, 435.8, 435.9 |
| ICD 10 | I63% I60%, I61%, I62% G45% |
| Bleeding | |
| ICD 9 | Transfusion code 99.00-99.07 AND a bleeding event code from the following: 535.01, 535.11, 535.21, 535.41, 535.51, 535.61, 537.83, 456.0, 456.2, 530.7, 530.82, 578.0, 455.2, 455.5, 455.8, 562.02, 562.03, 562.12, 562.13, 568.81, 569.3, 569.85, 578.1, 578.9, 593.81, 599.7, 623.8, 626.2, 626.6, 456.0, 456.2, 530.7, 530.82, 578.0, 455.2, 455.5, 455.8, 562.02, 562.03, 562.12, 562.13, 568.81, 569.3, 569.85, 578.1, 578.9, 593.81, 599.7, 623.8, 626.2, 626.6, 430, 431, 423.0, 459.0, 568.81, 784.7, 784.8, 786.3 OR 531.0% or 531.2% or 531.4% or i 531.6% or 532.0% or 532.2% or 532.4% or 532.6% or 533.0% or 533.2% or 33.4% or 533.6% or 534.0% or 534.2% or 534.4% or 534.6% or 432% or 852.0% or 852.2% or 852.4% or 853.0% or 853.1% or 719.1% |
| ICD 10 | Transfusion code [30233H1, 30233N1, 30233P1, 30243H1, 30243N1, 30243P1, 30233K1, 30233L1, 30243K1, 30243L1, 30283B1, 30233R1, 30243R1] AND a bleeding event code from the following: [K25.0, K25.2, K25.4, K25.6, K26.0, K26.2, K26.4, K26.6, K27.0, K27.2, K27.4, K27.6, K28.0, K28.2, K28.4, K28.6, K29.01, K29.21, K29.31, K29.41, K29.51, K29.61, K29.71, K29.81, K29.91, K31.811, I85.01, I85.11, K22.6, K22.8, K92.0, K55.21, K57.01, K57.11, K57.13, K57.21, K57.31, K57.33, K57.41, K57.51, K57.53, K57.81, K57.91, K57.93, K62.5, K64.0, K64.1, K64.2, K64.3, K64.4, K64.8, K66.1, K92.1, K92.2, N28.0, N89.8, N92.0, N92.1, R31.0, R31.1, R31.2, R31.9, I60.00, I60.01, I60.02, I60.10, I60.11, I60.12, I60.20, I60.21, I60.22, I60.30, I60.31, I60.32, I60.4, I60.50, I60.51, I60.52, I60.6, I60.7, I60.8, I60.9, I61.0, I61.1, I61.2, I61.3, I61.4, I61.5, I61.6, I61.8, I61.9, I62.00, I62.01, I62.02, I62.03, I62.1, I62.9, S06.340A, S06.341A, S06.342A, S06.343A, S06.344A, S06.345A, S06.346A, S06.347A, S06.348A, S06.349A, S06.350A, S06.351A, S06.352A, S06.353A, S06.354A, S06.355A, S06.356A, S06.357A, S06.358A, S06.359A, S06.360A, S06.361A, S06.362A, S06.363A, S06.364A, S06.365A, S06.366A, S06.367A, S06.368A, S06.369A, S06.369A, S06.4X0A, S06.4X1A, S06.4X2A, S06.4X3A, S06.4X4A, S06.4X5A, S06.4X6A, S06.4X7A, S06.4X8A, S06.4X9A, S06.5X0A, S06.5X1A, S06.5X2A, S06.5X3A, S06.5X4A, S06.5X5A, S06.5X6A, S06.5X7A, S06.5X8A, S06.5X9A, S06.6X0A, S06.6X1A, S06.6X2A, S06.6X3A, S06.6X4A, S06.6X5A, S06.6X6A, S06.6X7A, S06.6X8A, S06.6X9A, I31.2, R58, K66.1, M25.00, M25.011, M25.012, M25.019, M25.022, M25.021, M25.029, M25.031, M25.032, M25.039, M25.041, M25.042, M25.049, M25.051, M25.052, M25.059, M25.061, M25.062, M25.069, M25.071, M25.072, M25.074, M25.075, M25.073, M25.076, M25.08, M25.00, R04.0, R04.1, R04.2, R04.9, R04.81, R04.89] |
| Diabetes | |
| ICD 9 | 249.00, 249.01, 249.10, 249.11, 249.20, 249.21, 249.30, 249.31, 249.40, 249.41, 249.50, 249.51, 249.60, 249.61, 249.70, 249.71, 249.80, 249.81, 249.90, 249.91, 250.00, 250.01, 250.02, 250.03, 250.10, 250.11, 250.12, 250.13, 250.20, 250.21, 250.22, 250.23, 250.30, 250.31, 250.32, 250.33, 250.40, 250.41, 250.42, 250.43, 250.50, 250.51, 250.52, 250.53, 250.60, 250.61, 250.62, 250.63, 250.70, 250.71, 250.72, 250.73, 250.80, 250.81, 250.82, 250.83, 250.90, 250.91, 250.92, 250.93, 357.2, 362.01, 362.02, 362.03, 362.04, 362.05, 362.06, 366 |
| ICD 10 | E08.00, E08.01, E08.10, E08.11, E08.21, E08.22, E08.29, E08.311, E08.319, E08.321, E08.3211, E08.3212, E08.3213, E08.3219, E08.329, E08.3291, E08.3292, E08.3293, E08.3299, E08.331, E08.3311, E08.3312, E08.3313, E08.3319, E08.339, E08.3391, E08.3392, E08.3393, E08.3399, E08.341, E08.3411, E08.3412, E08.3413, E08.3419, E08.349, E08.3491, E08.3492, E08.3493, E08.3499, E08.351, E08.3511, E08.3512, E08.3513, E08.3519, E08.3521, E08.3522, E08.3523, E08.3529, E08.3531, E08.3532, E08.3533, E08.3539, E08.3541, E08.3542, E08.3543, E08.3549, E08.3551, E08.3552, E08.3553, E08.3559, E08.359, E08.3591, E08.3592, E08.3593, E08.3599, E08.36, E08.37X1, E08.37X2, E08.37X3, E08.37X9, E08.39, E08.40, E08.41, E08.42, E08.43, E08.44, E08.49, E08.51, E08.52, E08.59, E08.610, E08.618, E08.620, E08.621, E08.622, E08.628, E08.630, E08.638, E08.641, E08.649, E08.65, E08.69, E08.8, E08.9, E09.00, E09.01, E09.10, E09.11, E09.21, E09.22, E09.29, E09.311, E09.319, E09.321, E09.3211, E09.3212, E09.3213, E09.3219, E09.329, E09.3291, E09.3292, E09.3293, E09.3299, E09.331, E09.3311, E09.3312, E09.3313, E09.3319, E09.339, E09.3391, E09.3392, E09.3393, E09.3399, E09.341, E09.3411, E09.3412, E09.3413, E09.3419, E09.349, E09.3491, E09.3492, E09.3493, E09.3499, E09.351, E09.3511, E09.3512, E09.3513, E09.3519, E09.3521, E09.3522, E09.3523, E09.3529, E09.3531, E09.3532, E09.3533, E09.3539, E09.3541, E09.3542, E09.3543, E09.3549, E09.3551, E09.3552, E09.3553, E09.3559, E09.359, E09.3591, E09.3592, E09.3593, E09.3599, E09.36, E09.37X1, E09.37X2, E09.37X3, E09.37X9, E09.39, E09.40, E09.41, E09.42, E09.43, E09.44, E09.49, E09.51, E09.52, E09.59, E09.610, E09.618, E09.620, E09.621, E09.622, E09.628, E09.630, E09.638, E09.641, E09.649, E09.65, E09.69, E09.8, E09.9, E10.10, E10.11, E10.21, E10.22, E10.29, E10.311, E10.319, E10.321, E10.3211, E10.3212, E10.3213, E10.3219, E10.329, E10.3291, E10.3292, E10.3293, E10.3299, E10.331, E10.3311, E10.3312, E10.3313, E10.3319, E10.339, E10.3391, E10.3392, E10.3393, E10.3399, E10.341, E10.3411, E10.3412, E10.3413, E10.3419, E10.349, E10.3491, E10.3492, E10.3493, E10.3499, E10.351, E10.3511, E10.3512, E10.3513, E10.3519, E10.359, E10.36, E10.37X1, E10.37X2, E10.37X3, E10.37X9, E10.39, E10.40, E10.41, E10.42, E10.43, E10.44, E10.49, E10.51, E10.52, E10.59, E10.610, E10.618, E10.620, E10.621, E10.622, E10.628, E10.630, E10.638, E10.641, E10.649, E10.65, E10.69, E10.8, E10.9, E11.00, E11.01, E11.10, E11.11, E11.21, E11.22, E11.29, E11.311, E11.319, E11.321, E11.3211, E11.3212, E11.3213, E11.3219, E11.329, E11.3291, E11.3292, E11.3293, E11.3299, E11.331, E11.3311, E11.3312, E11.3313, E11.3319, E11.339, E11.3391, E11.3392, E11.3393, E11.3399, E11.341, E11.3411, E11.3412, E11.3413, E11.3419, E11.349, E11.3491, E11.3492, E11.3493, E11.3499, E11.351, E11.3511, E11.3512, E11.3513, E11.3519, E11.3521, E11.3522, E11.3523, E11.3529, E11.3531, E11.3532, E11.3533, E11.3539, E11.3541, E11.3542, E11.3543, E11.3549, E11.3551, E11.3552, E11.3553, E11.3559, E11.359, E11.3591, E11.3592, E11.3593, E11.3599, E11.36, E11.37X1, E11.37X2, E11.37X3, E11.37X9, E11.39, E11.40, E11.41, E11.42, E11.43, E11.44, E11.49, E11.51, E11.52, E11.59, E11.610, E11.618, E11.620, E11.621, E11.622, E11.628, E11.630, E11.638, E11.641, E11.649, E11.65, E11.69, E11.8, E11.9, E13.00, E13.01, E13.10, E13.11, E13.21, E13.22, E13.29, E13.311, E13.319, E13.321, E13.3211, E13.3212, E13.3213, E13.3219, E13.329, E13.3291, E13.3292, E13.3293, E13.3299, E13.331, E13.3311, E13.3312, E13.3313, E13.3319, E13.339, E13.3391, E13.3392, E13.3393, E13.3399, E13.341, E13.3411, E13.3412, E13.3413, E13.3419, E13.349, E13.3491, E13.3492, E13.3493, E13.3499, E13.351, E13.3511, E13.3512, E13.3513, E13.3519, E13.3521, E13.3522, E13.3523, E13.3529, E13.3531, E13.3532, E13.3533, E13.3539, E13.3541, E13.3542, E13.3543, E13.3549, E13.3551, E13.3552, E13.3553, E13.3559, E13.359, E13.36, E13.39, E13.40, E13.41, E13.42, E13.43, E13.44, E13.49, E13.51, E13.52, E13.59, E13.610, E13.618, E13.620, E13.621, E13.622, E13.628, E13.630, E13.638, E13.641, E13.649, E13.65, E13.69, E13.8, E13.9 |
| Hypertension | |
| ICD 9 | 401.xx, 402.xx, 403.xx, 404.xx |
| ICD 10 | I10.%, I11.%, I12.%, I13.%, H35.03%, I67.4 |
| Cardiac arrest | |
| ICD 9 | 427.5, 798, 798.1 |
| ICD 10 | I46.0, I46.2, I46.8, I46.9 |
| Atrial fibrillation | |
| ICD 9 | 427.31 |
| ICD 10 | I48.0, I48.1, I48.11, I48.19, I48.2, I48.21, I48.91 |
| Ventricular arrhythmia | |
| ICD 9 | 427.1, 427.4, 427.41, 427.42, 427.9, |
| ICD 10 | I47.2, I49.0, I49.01 |
| Chronic Kidney Disease | |
| ICD 9 | 016.00, 016.01, 016.02, 016.03, 016.04, 016.05, 016.06, 095.4, 189.0, 189.9, 223.0, 236.91, 249.40, 249.41, 250.40, 250.41, 250.42, 250.43, 271.4, 274.10, 283.11, 403.01, 403.11, 403.91, 404.02, 404.03, 404.12, 404.13, 404.92, 404.93, 440.1, 442.1, 572.4, 580.0, 580.4, 580.81, 580.89, 580.9, 581.0, 581.1, 581.2, 581.3, 581.81, 581.89, 581.9, 582.0, 582.1, 582.2, 582.4, 582.81, 582.89, 582.9, 583.0, 583.1, 583.2, 583.4, 583.6, 583.7, 583.81, 583.89, 583.9, 584.5, 584.6, 584.7, 584.8, 584.9, 585.1, 585.2, 585.3, 585.4, 585.5, 585.6, 585.9, 586, 587, 588.0, 588.1, 588.81, 588.89, 588.9, 591, 753.12, 753.13, 753.14, 753.15, 753.16, 753.17, 753.19, 753.20, 753.21, 753.22, 753.23, 753.29, 794 |
| ICD 10 | A18.11, A52.75, B52.0, C64.1, C64.2, C64.9, C68.9, D30.00, D30.01, D30.02, D41.00, D41.01, D41.02, D41.10, D41.11, D41.12, D41.20, D41.21, D41.22, D59.3, E08.21, E08.22, E08.29, E08.65, E09.21, E09.22, E09.29, E10.21, E10.22, E10.29, E10.65, E11.21, E11.22, E11.29, E11.65, E13.21, E13.22, E13.29, E74.8, I12.0, I12.9, I13.0, I13.10, I13.11, I13.2, I70.1, I72.2, K76.7, M10.30, M10.311, M10.312, M10.319, M10.321, M10.322, M10.329, M10.331, M10.332, M10.339, M10.341, M10.342, M10.349, M10.351, M10.352, M10.359, M10.361, M10.362, M10.369, M10.371, M10.372, M10.379, M10.38, M10.39, M32.14, M32.15, M35.04, N00.0, N00.1, N00.2, N00.3, N00.4, N00.5, N00.6, N00.7, N00.8, N00.9, N00.A, N01.0, N01.1, N01.2, N01.3, N01.4, N01.5, N01.6, N01.7, N01.8, N01.9, N01.A, N02.0, N02.1, N02.2, N02.3, N02.4, N02.5, N02.6, N02.7, N02.8, N02.9, N02.A, N03.0, N03.1, N03.2, N03.3, N03.4, N03.5, N03.6, N03.7, N03.8, N03.9, N03.A, N04.0, N04.1, N04.2, N04.3, N04.4, N04.5, N04.6, N04.7, N04.8, N04.9, N04.A, N05.0, N05.1, N05.2, N05.3, N05.4, N05.5, N05.6, N05.7, N05.8, N05.9, N05.A, N06.0, N06.1, N06.2, N06.3, N06.4, N06.5, N06.6, N06.7, N06.8, N06.9, N06.A, N07.0, N07.1, N07.2, N07.3, N07.4, N07.5, N07.6, N07.7, N07.8, N07.9, N07.A, N08, N13.1, N13.2, N13.30, N13.39, N14.0, N14.1, N14.2, N14.3, N14.4, N15.0, N15.8, N15.9, N16, N17.0, N17.1, N17.2, N17.8, N17.9, N18.1, N18.2, N18.3, N18.30, N18.31, N18.32, N18.4, N18.5, N18.6, N18.9, N19, N25.0, N25.1, N25.81, N25.89, N25.9, N26.1, N26.9, Q61.02, Q61.11, Q61.19, Q61.2, Q61.3, Q61.4, Q61.5, Q61.8, Q62.0, Q62.2, Q62.10, Q62.11, Q62.12, Q62.31, Q62.32, Q62.39, R94.4 |
| Valvular Heart Disease | |
| ICD 9 | 093.2, 394.x-397.x, 424.x, 746.3-746.6, V42.2, V43.3 |
| ICD 10 | A52.0, I05.x-I08.x, I09.1, I09.8, I34.x-I39.x, Q23.OQ23.3, Z95.2, Z95.4 |
| Peripheral artery disease | |
| ICD 9 | 093.0, 437.3, 440.x, 441.x, 443.1-443.9, 447.1, 557.1 557.9, V43.4 |
| ICD 10 | I70.x, I71.x, I73.1, I73.8, I73.9, I77.1, I79.0, I79.2, K55.1, K55.8, K55.9, Z95.8, Z95.9 |
| COPD | |
| ICD 9 | 490, 491.0, 491.1, 491.20, 491.21, 491.22, 491.8, 491.9, 492.0, 492.8, 494.0, 494.1, 496 |
| ICD 10 | J40, J41.0, J41.1, J41.8, J42, J43.0, J43.1, J43.2, J43.9, J44.0, J44.1, J44.9, J47.0, J47.1, J47.9, J98.2, J98.3 |
| Coagulopathy | |
| ICD 9 | 286.x, 287.1, 287.3-287.5 |
| ICD 10 | D65-D68.x, D69.1, D69.3-D69.6 |
| Obesity | |
| ICD 9 | 278.0 |
| ICD 10 | E66.x |
| Cachexia | |
| ICD 9 | 260.x-263.x, 783.2, 799.4 |
| ICD 10 | E40.x-E46.x, R63.4, R64 |
| Depression | |
| ICD 9 | 296.20, 296.21, 296.22, 296.23, 296.24, 296.25, 296.26, 296.30, 296.31, 296.32, 296.33, 296.34, 296.35, 296.36, 296.51, 296.52, 296.53, 296.54, 296.55, 296.56, 296.60, 296.61, 296.62, 296.63, 296.64, 296.65, 296.66, 296.89, 298.0, 300.4, 309.1, 311 |
| ICD 10 | F31.30, F31.31, F31.32, F31.4, F31.5, F31.60, F31.61, F31.62, F31.63, F31.64, F31.75, F31.76, F31.77, F31.78, F31.81, F32.0, F32.1, F32.2, F32.3, F32.4, F32.5, F32.9, F33.0, F33.1, F33.2, F33.3, F33.40, F33.41, F33.42, F33.8, F33.9, F34.1, F43.21, F43.23 |
| Substance abuse | |
| ICD 9 | 292.x, 304.x, 305.2-305.9, V65.42 |
| ICD 10 | F11.x-F16.x, F18.x, F19.x, Z71.5. Z72.2 |
| Ischemic cardiomyopathy | |
| ICD 9 | I20.0, I20.1, I20.8, I20.9, I21.01, I21.02, I21.09, I21.11, I21.19, I21.21, I21.29, I21.3, I21.4, I21.A1, I21.A9, I22.0, I22.1, I22.2, I22.8, I22.9, I23.0, I23.1, I23.2, I23.3, I23.4, I23.5, I23.6, I23.7, I23.8, I24.0, I24.1, I24.8, I24.9, I25.10, I25.110, I25.111, I25.118, I25.119, I25.2, I25.3, I25.41, I25.42, I25.5, I25.6, I25.700, I25.701, I25.708, I25.709, I25.710, I25.711, I25.718, I25.719, I25.720, I25.721, I25.728, I25.729, I25.730, I25.731, I25.738, I25.739, I25.750, I25.751, I25.758, I25.759, I25.760, I25.761, I25.768, I25.769, I25.790, I25.791, I25.798, I25.799, I25.810, I25.811, I25.812, I25.82, I25.83, I25.84, I25.89, I25.9 |
| ICD 10 | I20.0, I20.1, I20.8, I20.9, I21.01, I21.02, I21.09, I21.11, I21.19, I21.21, I21.29, I21.3, I21.4, I21.A1, I21.A9, I22.0, I22.1, I22.2, I22.8, I22.9, I23.0, I23.1, I23.2, I23.3, I23.4, I23.5, I23.6, I23.7, I23.8, I24.0, I24.1, I24.8, I24.9, I25.10, I25.110, I25.111, I25.118, I25.119, I25.2, I25.3, I25.41, I25.42, I25.5, I25.6, I25.700, I25.701, I25.708, I25.709, I25.710, I25.711, I25.718, I25.719, I25.720, I25.721, I25.728, I25.729, I25.730, I25.731, I25.738, I25.739, I25.750, I25.751, I25.758, I25.759, I25.760, I25.761, I25.768, I25.769, I25.790, I25.791, I25.798, I25.799, I25.810, I25.811, I25.812, I25.82, I25.83, I25.84, I25.89, I25.9 |
