# Supplementary material for: National Trends for Temporary Mechanical Circulatory Support Utilization in Patients With Cardiogenic Shock From Decompensated Chronic Heart Failure: Incidence, Predictors, Outcomes, and Cost
Source: J Soc Cardiovasc Angiogr Interv. 2023 Dec 4;2(6Part B):101177. doi: 10.1016/j.jscai.2023.101177 (PMC11307713; doi:10.1016/j.jscai.2023.101177)
Supplement: Supplemental Table S2 [file mmc4.docx]

Supplemental Table 2: Predictors of temporary mechanical circulatory support device selection in ischemic and non-ischemic cardiomyopathy patients stratified by use of ICD 9 vs. ICD 10 codes

1. ICD 9 codes

| Variable | Ischemic cardiomyopathy*  AOR (95% CI) | | Non ischemic cardiomyopathy*  AOR (95% CI) | |
| --- | --- | --- | --- | --- |
|  | ECMO | Impella | ECMO | Impella |
| Female sex | 1.71 (1.07-2.74) | 0.35 (0.17-0.73) | 1.12 (0.70-1.80) | 0.83 (0.39-1.77) |
| Race (White) | 0.90 (0.44-1.85) | 1.24 (0.51-3.00) | 0.99 (0.48-2.05) | 1.23 (0.38-3.96) |
| Commercial Insurance | 0.88 (0.53-1.46) | 0.87 (0.50-1.51) | 2.59 (1.40-4.80) | 4.70 (1.35-16.37) |
| Hispanic ethnicity | 0.89 (0.30-2.58) | 1.86 (0.63-5.51) | 3.13 (1.14-8.61) | 0.71 (0.07-7.40) |
| Diabetes Mellitus | 0.59 (0.36-0.98) | 1.31 (0.75-2.28) | 0.85 (0.47-1.53) | 0.58 (0.22-1.54) |
| Hypertension | 0.98 (0.53-1.80) | 1.10 (0.52-2.19) | 0.66 (0.40-1.09) | 0.96 (0.44-2.10) |
| Acute Kidney Injury | 1.63 (1.00-2.65) | 3.23 (1.84-5.66) | 2.50 (1.53-4.08) | 1.85 (0.86-3.96) |
| Alcohol/Substance Abuse Disorder | 0.60 (0.19-1.89) | 0.65 (0.18-2.32) | 0.90 (0.39-2.06) | 0.50 (0.11-2.38) |
| Valvular Heart Disease | 0.85 (0.59-1.20) | 1.01 (0.81-1.47) | 0.34 (0.20-0.56) | 0.36 (0.17-0.77) |
| Peripheral Arterial Disease | 0.48 (0.36-0.65) | 0.51 (0.39 – 0.66) | 1.42 (0.76-2.64) | 2.56 (1.06-6.19) |
| Cachexia | 1.24 (0.77-2.01) | 0.59 (0.35-1.00) | 1.74 (1.09-2.78) | 1.08 (0.52-2.26) |
| Coagulopathy | 2.05 (1.24-3.37) | 0.93 (0.49-1.75) | 1.93 (0.87-4.28) | 0.64 (0.33-1.24) |
| Obesity | 1.54 (0.81-2.93) | 1.09 (0.54-2.21) | 0.64 (0.33-1.24) | 0.79 (0.29-2.18) |
| CKD | 2.70 (1.58-4.74) | 0.95 (0.44-2.05) | 2.09 (1.18-3.70) | 0.65 (0.20-2.10) |
| Blood Loss Anemia | 1.55 (0.67-3.61) | 0.80 (0.25-2.52) | 1.74 (0.45-6.72) | 2.37 (0.21-26.37) |
| *Intra-aortic balloon pump as reference  Abbreviations: AOR=Adjusted Odds Ratios ; CI – confidence interval; CKD- chronic kidney disease; COPD – chronic obstructive pulmonary disease. | | | | |

1. ICD 10 codes

| Variable | Ischemic cardiomyopathy*  AOR (95% CI) | | Non ischemic cardiomyopathy*  AOR (95% CI) | |
| --- | --- | --- | --- | --- |
|  | ECMO | Impella | ECMO | Impella |
| Female sex | 1.05 (0.72-1.54) | 0.97 (0.71-1.33) | 1.29 (0.78-2.14) | 1.05 (0.56-1.96) |
| Commercial Insurance | 1.12 (0.76-1.66) | 0.68 (0.48-0.95) | 1.73 (0.94-3.20) | 0.69 (0.32-1.49) |
| Race (White) | 0.87 (0.56-1.35) | 1.00 (0.69-1.44) | 1.32 (0.66-2.63) | 1.10 (0.50-2.45) |
| Hispanic ethnicity | 0.82 (0.41-1.65) | 0.54 (0.29-1.03) | 1.63 (0.52-5.12) | 1.02 (0.25-4.16) |
| Diabetes Mellitus | 0.96 (0.68-1.36) | 1.10 (0.83-1.45) | 0.63 (0.35-1.13) | 2.11 (1.07-4.15) |
| Hypertension | 0.81 (0.48-1.35) | 1.01 (0.65-1.57) | 0.54 (0.31-0.94) | 0.30 (0.15-0.59) |
| Acute Kidney Injury | 1.43 (0.97-2.10) | 0.89 (0.66-1.21) | 1.57 (0.89-2.78) | 2.60 (1.25-5.39) |
| Alcohol/Substance Abuse Disorder | 0.83 (0.48-1.44) | 1.29 (0.81-2.06) | 1.77 (0.94-3.36) | 1.85 (0.85-4.03) |
| Atrial Fibrillation | 0.85 (0.60-1.19) | 0.86 (0.66-1.14) | 0.55 (0.34-0.91) | 0.35 (0.19-0.67) |
| Cardiac arrest | 3.00 (1.83-4.94) | 1.33 (0.79-2.27) | 1.42 (0.73-2.75) | 0.91 (0.38-2.14) |
| Ventricular Tachycardia | 0.64 (0.44-0.93) | 0.96 (0.70-1.30) | 0.76 (0.44-1.30) | 1.41 (0.74-2.70) |
| Valvular Heart Disease | 0.53 (0.37-0.76) | 0.58 (0.43-0.79) | 0.28 (0.17-0.46) | 0.44 (0.24-0.83) |
| Peripheral Arterial Disease | 0.78 (0.54-1.14) | 1.07 (0.78-1.47) | 1.00 (0.59-1.64) | 2.07 (1.09-3.93) |
| Cachexia | 1.14 (0.80-1.61) | 0.78 (0.58-1.04) | 2.44 (1.36-4.39) | 1.06 (0.48-2.30) |
| Coagulopathy | 2.19 (1.50-3.21) | 0.85 (0.61-1.20) | 2.43 (1.42-4.16) | 1.40 (0.72-2.72) |
| Obesity | 1.03 (0.71-1.48) | 0.91 (0.67-1.24) | 0.66 (0.39-1.13) | 0.54 (0.27-1.06) |
| CKD | 0.85 (0.57-1.28) | 0.69 (0.48-1.00) | 1.22 (0.67-2.24) | 0.85 (0.40-1.82) |
| Blood Loss Anemia | 1.43 (0.73-2.81) | 0.44 (0.19-1.02) | 2.98 (0.82-10.84) | 1.74 (0.33-9.25) |
| *Intra-aortic balloon pump as reference  Abbreviations: AOR=Adjusted Odds Ratios ; CI – confidence interval; CKD- chronic kidney disease; COPD – chronic obstructive pulmonary disease. | | | | |
